# The effect of becoming a Fistula Advocate on the recovery of women with Obstetric Fistula in Sierra Leone: A qualitative study

**DOI:** 10.1371/journal.pgph.0000765

**Published:** 2023-04-12

**Authors:** Zoë Vowles, Regina Bash-Taqi, Alusine Kamara, Mabel Kuteh, Sergio A. Silverio, Ibrahim Turay, Stephen Peckham

**Affiliations:** 1 Department of Women & Children’s Health, School of Life Course & Population Sciences, King’s College London, London, United Kingdom; 2 St Thomas’ Hospital, Guy’s and St Thomas’ NHS Foundation Trust, London, United Kingdom; 3 Department of Health Services Research and Policy, Faculty of Public Health and Policy, The London School of Hygiene and Tropical Medicine, London, United Kingdom; 4 Institute for Development, Freetown, Sierra Leone; 5 Health Poverty Action, Freetown, Sierra Leone; 6 The Centre for Health Service Studies, University of Kent, Canterbury, United Kingdom; PLOS: Public Library of Science, UNITED STATES

## Abstract

Engaging women affected by Obstetric Fistula as advocates has been proposed as an effective strategy to raise awareness of the condition. Limited literature exists on the experience of those who become advocates. A model of community education, in Sierra Leone, trained women affected by Obstetric Fistula to become volunteer Fistula Advocates. This study explored Advocates’ perception of their role and its influence on their recovery and reintegration. This was a qualitative study, undertaken in Sierra Leone, collecting data from 7 Fistula Advocates and 3 Key Informants (with roles in either clinical or outreach care for women with Obstetric Fistula or training and supervision of Advocates), using semi-structured interviews. Data was subject to a thematic analysis and related to a conceptual framework for mental health recovery. Intrinsic factors motivating Advocates to undertake this role were influenced by psycho-social support received and the possibility for financial independence. Advocates used personal stories in their work to define a new identity, change perceptions and reduce stigma. Benefits associated with the interactions and relationships created through providing and receiving peer support were voiced. Surgical treatment was described as an important factor influencing recovery. The Advocates said economic empowerment helped recovery and reintegration, and the voluntary nature of the Advocate role limited the impact of this. Overall Advocates perceived their role positively, reporting psychological, social, and economic benefits. The complexities of recovery from Obstetric Fistula were highlighted and connections drawn between the treatment of physical symptoms, the socio-cultural context and mental health recovery. They described the role positively influencing existing relationships and initiating supportive, empowering social interactions between women affected by Obstetric Fistula and with Non-Governmental Organisation staff and community members. The study offers insights into the potential for community-based approaches to facilitate access to treatment for sensitive and stigmatising health problems and support recovery.

## 1. Introduction

### 1.1 Obstetric Fistula and the global context

Obstetric Fistula is a childbirth injury resulting in life altering physical and psychological morbidity. Accurate and reliable data on the burden of Obstetric Fistula is limited and it is estimated that between 500,000 and 2 million women are living with Obstetric Fistula globally [1, 2]. The majority of cases develop as a consequence of obstructed labour [3], leading to the formation of an abnormal opening between the vagina and either the bladder, rectum or both [4, 5]. Women who survive and then develop a fistula are left with uncontrollable leaking of urine and/or faeces from the vagina [5, 6]. Although presenting primarily as a medical complication of childbirth, Obstetric Fistula has complex causes. Social and cultural factors such as inequalities experienced by women and girls, child marriage and adolescent pregnancy coupled with the quality and availability of reproductive health services contribute to continued high rates of Obstetric Fistula [2, 6].

The continued incidence of Obstetric Fistula highlights the inequities experienced by women in low resource countries; since it has been almost eradicated in high income countries with well-developed health services [7, 8]. Obstetric Fistula is preventable when women have access to high quality, comprehensive maternal health services [9]. Obstetric Fistula disproportionately affects the poorest women, whose voices are scarcely heard, with the burden of disease resting predominantly upon poor and marginalised women and girls in Sub-Saharan Africa and Asia [6].

The need to support women and girls living with Obstetric Fistula, in addition to preventing the condition occurring, has been identified by research undertaken in Sierra Leone which aimed to shed light on the experiences of women suffering from the condition [10]. The 2030 Agenda for Sustainable Development/ Sustainable Development Goals (SDGs) provides an opportunity to advance gender equality and promote health and wellbeing for all [11, 12]. The principle to ‘leave no one behind’ is at the heart of the SDGs [12, 13]. Ending the needless morbidity and suffering associated with the continued incidence of Obstetric Fistula is key to ensuring women and girls with Obstetric Fistula do not get ‘left behind’ [12]. The United Nations (UN) Secretary General’s 2018 report on fistula [9] identified ending Obstetric Fistula as crucial to achieving many of the SDGs; particularly those relating to poverty, health, education, gender equality and inequalities. This report was followed by the UN resolution A/HRC/RES/39/10 on Preventable maternal mortality and morbidity and human rights in humanitarian settings, which was adopted by the Human Rights Council and calls for accelerated progress to end fistula with achievement of the SDGs within a decade [14].

The impact of Obstetric Fistula upon the lives of women affected is profound and debilitating; physical problems, if untreated, can result in premature mortality [4, 5, 9]. In addition to the serious physical consequences women face, there are numerous and enduring psychological and social implications [15–17]. The uncontrollable leaking of urine, constant wetness and odour mean women may experience stigma, discrimination or internalised feelings of shame, leading to depression and isolation [6, 15]. Furthermore, 90% of women will experience the sadness and loss of giving birth to a baby who is either stillborn or dies soon after birth [6]. Obstetric Fistula can also negatively impact household relationships and social networks [18].

Obstetric Fistula limits women’s ability to undertake activities to generate income, creates deepening poverty, economic dependence and increases their susceptibility to violence and abuse [4–6]. The myriad of physical, social, and psychological consequences translates into an increased risk of mental disorder, depression and suicide among women affected [9, 19–21].

The need to develop recovery strategies which meet the physical, psychosocial and economic needs of women affected and empower them to actively participate in the development of prevention and treatment programmes has been highlighted in the country-level needs assessments conducted in 29 countries in the Campaign to End Fistula [6]. Offering women affected by Obstetric Fistula the choice to undertake advocacy activities to raise awareness in communities of maternal and newborn care, safe birth, Obstetric Fistula prevention and treatment and dispel myths associated with the condition has been described an effective strategy for Obstetric Fistula prevention and treatment by the World Health Organization [5]. The role of Obstetric Fistula survivors in raising awareness of fistula prevention and treatment has been described in the 2014 and 2018 United Nations General Assembly Secretary-General Reports on ending Obstetric Fistula [9, 22]. A study exploring women’s experiences with fistula repair surgery in Malawi identified that women who have received successful treatment may serve as effective role models, illustrating the benefits of treatment in their community [23]. Furthermore, it has been suggested by the Campaign to End Fistula country-level needs assessments that the advocacy role has the potential to facilitate empowerment and reintegration of survivors [6]. However, there is limited evidence on the experiences of women affected by Obstetric Fistula, who become Advocates. This paper reports on an exploratory study into the experiences of Advocates, how they perceive their role, and its influence on their recovery.

### 1.2 Theoretical approach

This study explored a concept of recovery encompassing social reintegration and psychological well-being [24]; as the serious psychological and social effects of Obstetric Fistula on women including the associated risks of mental illness and suicide are well documented [20, 25, 26]. Therefore, literature related to mental health recovery is most theoretically relevant to apply to recovery from Obstetric Fistula. In 2007, Onken suggested using an ecological framework to conceptualise mental health recovery; proposing recovery relates to individual recognition that change is possible and systemic change within the individual’s environment and more importantly, the impact of interactions between the two [27]. This framework conceptualises recovery as both overcoming physical symptoms, stigma and social exclusion resulting from the illness [27]. Onken’s framework outlines four elements of recovery [27]. ‘*Person-Centered Elements’* focusing on aspects of personal motivation; including the ability of the individual to make autonomous decisions and find meaning in their daily life, which is also influenced by their social context. ‘*Re-authoring’* refers to an individual’s ability to re-define themselves, move forward and achieve personal goals following a traumatic experience through developing, taking ownership of and telling their own story. ‘*Exchange-Centered Elements’* describe the ability of the individual to participate actively and meaningfully within their community, building on the concept of re-authoring; utilising the positive identity gained during that process to forge a new, powerful social role. Peer support is identified as a mechanism to create a positive social identity. ‘*Community-Centred Elements’* relate to the presence of an environment which provides individuals with both their basic needs and the opportunity of nurturing relationships supporting reintegration into community life; without which recovery is not possible.

Data and analysis were compared to Onken’s ecological framework [27] to explore associations between themes; and with those identified in existing literature relating to recovery from Obstetric Fistula, specifically the complex relationship between individual recovery and the social environment.

## 2. Methods

### 2.1 Ethical considerations

Ethical clearance for this study was granted by both the Sierra Leone Ethics and Scientific Review Committee (SLESRC) and the London School of Hygiene and Tropical Medicine (LSHTM) Ethics Committee (Application number: 011/30).

### 2.2 Setting: Obstetric Fistula in Sierra Leone

At the time of the study, Sierra Leone was ranked 180 out of 187 countries with comparable data by the United Nations Development Programme (UNDP) Human Development Index [28]. The maternal mortality ratio was 890 per 100,000 live births, among the highest in the world [29].

Good quality data on the prevalence and incidence of Obstetric Fistula in Sierra Leone is limited [30, 31]. The inclusion of Sierra Leone as one of the focal countries in the United Nations Population Fund’s (UNFPA) global *‘Campaign To End Fistula’* indicates the magnitude of the disease burden in Sierra Leone [6, 32]. The researcher (ZV) attended Sierra Leone’s first National Conference on Obstetric Fistula in April 2012. At the meeting, the need to develop holistic treatment strategies, involving women affected by Obstetric Fistula as advocates for prevention and treatment, was emphasised. However, there is little known about the role of advocacy in survivors’ recovery and reintegration.

The Fistula Prevention Project (FPP) and study setting was Northern Bombali District. At the time of the study according to the 2008 SLDHS, women in the Northern region reported the highest rates of no education (77%) and illiteracy (84%) in the country [33]. Employment of women was high in the region [33], consisting predominantly of small-scale, informal agriculture [34], but control over earnings remained low. Of the women in employment, 46% reported use of earnings was mainly decided by their husband [33]. At the time of the study, this region had some of the poorest health indicators for women in the country, including the highest rates of teenage pregnancy (32.6%) and lowest rates of knowledge and use of contraception, (66% and 4% respectively). Only 27% of births in this region were attended by a Skilled Birth Attendant, compared to at least 50% in all other regions [33].

The FPP launched in 2008 in the Northern Bombali District in Sierra Leone. The aim of the project was to train women affected by Obstetric Fistula, who previously received surgical treatment, to become volunteer Fistula Advocates (hereafter, referred to as Advocates). This was undertaken in collaboration with Health Poverty Action (HPA), a Non-Governmental Organization (NGO), working in Sierra Leone since 2005. The purpose of the role was to deliver innovative community education to raise awareness of Obstetric Fistula prevention, actively look for other women living with Obstetric Fistula, provide peer support and facilitate referral for treatment; whilst simultaneously building Advocates’ confidence and skills.

The project provided an initial two-day training covering causes and prevention of Obstetric Fistula, how to refer patients for treatment, communication and advocacy skills and record keeping. A key material used during the training and by Advocates, was a picture book developed by HPA in partnership with Government of Sierra Leone Ministry of Health and Sanitation, Mercy Ships Fistula Centre (Now Aberdeen Women’s Centre), West African Fistula Centre, and women affected by Obstetric Fistula. The picture book illustrated the key messages around Obstetric Fistula prevention and treatment using the story of ‘Fatu’, a young girl who develops Obstetric Fistula following prolonged labour.

The Advocates received monthly supportive supervision visits from HPA field officers, participated in bi-annual Advocate Network meetings and received a token monetary sum (Le 20,000/ £3 per month) for their participation. Funding and programmatic changes to the FPP, proposed after this study was developed, were in progress when interviews were conducted.

### 2.3 Sampling

This paper draws on data from in depth interviews with Fistula Advocates (n = 7) participating in the FPP and Key Informants (KI; n = 3), Key Informants in this study were people who have first-hand or expert knowledge and experience of either care of women with Obstetric Fistula, Obstetric Fistula recovery or Advocate training and programming [35]. The KIs had roles in either clinical or outreach care for women with Obstetric Fistula or training and supervision of Advocates. Purposive or criteria sampling was used to identify a sample of 7 Advocates and 3 KIs with a range of experiences.

This sample size is in keeping with other studies on the experiences of women living with Obstetric Fistula [36]. The sample was chosen through discussions with project staff, thinking critically about participants most likely to provide rich data to answer the research question effectively [37]. Women with a range of experiences of becoming an Advocate and recovering from Obstetric Fistula including those of different ages, who had lived with Obstetric Fistula for different durations, with and without living children were included in the study. The KIs were selected for their knowledge and experience of either Obstetric Fistula, Obstetric Fistula recovery or Advocate training and programming.

### 2.4 Characteristics of participants

The characteristics of participants are summarised in Table 1. All participants invited to take part in the research agreed to participate. No Advocates were able to report their age. All Advocates reported being married as young girls, becoming pregnant soon after; six out of the seven Advocates interviewed developed Obstetric Fistula during their first birth. Number of years spent with Obstetric Fistula prior to repair surgery ranged from <1 to > 20, demonstrating the age diversity of participants. All Advocates reported no education, none were literate or able to write their name. All Advocates had previously received surgical treatment; only one requiring more than one operation to be successfully repaired. To preserve anonymity limited information is provided on the characteristics of KIs due to the small number of people working on Obstetric Fistula in Sierra Leone.

**Table 1:** Participant characteristics.

Participant	Education	Marital Status	Children Alive	Years with Obstetric Fistula prior to repair
FA1	None	Married	2 (After repair)	3
FA2	None	Divorced after Obstetric Fistula Now re-married	None	>20
FA3	None	Married	None	Exact years unknown–reported many years with Obstetric Fistula
FA4	None	Married	2 (After Obstetric Fistula)	6
FA5	None	Married	2 alive and 2 died	<1
FA6	None	Divorced after Obstetric Fistula Now re-married	None	Exact years unknown–reported many years with Obstetric Fistula

### 2.5 Data collection

Interviews were conducted in 2012 face to face by the researcher (ZV) and a research assistant from HPA (AK) who assisted with translation. Interviews were conducted in a location chosen by the participant. All but one interview with Advocates occurred in or near their homes, in a setting where they felt comfortable to talk to the researcher; one interview took place in a private room in HPA field office. Interviews with KIs took place in a private room in their workplace. Each semi-structured interview lasted approximately 45–60 minutes. During part of the interview participants were asked questions relating to their experience of living with Obstetric Fistula prior to and post repair surgery. This is a sensitive topic, as women living with Obstetric Fistula may have experienced stigma and social exclusion. The information given made clear that agreeing to participate did not mean requirement to answer any question found difficult or distressing. Women reporting health concerns were advised to seek advice from the Peripheral Health Unit or Hospital according to their location and symptoms.

Four interviews were conducted in Krio; the researcher (ZV) (who had basic Krio language skills) worked alongside the research assistant (AK) who assisted with translation or clarification when required. Three participants did not speak Krio or preferred to be interviewed in their local language. Therefore, two were in Temne and one in Limba.

A field diary of daily activities, informal conversations with participants, meetings with HPA field and project staff and notes from the National Conference was documented throughout the research process. This process enabled ideas to be captured adding depth and context to the interview data [38].

All the Advocates interviewed were illiterate; therefore, information leaflets were translated verbally into either Krio or their local language. All participants were given the opportunity to ask questions, decline to participate and finally asked to provide formal written consent prior to participation in the study, (indicated with a thumbprint if unable to write their name which is common practice in Sierra Leone).

### 2.6 Data management and analysis

Interviews conducted in Temne or Limba were translated verbally into English immediately after the interview and transcribed in English; translation was discussed between the first author (ZV) and third author (AK) (fluent in Temne, Limba, Krio and English). Interviews conducted in Krio, were translated, and transcribed by the first author (ZV) with clarification sought from the third author (AK) when required. Data was managed using NVivo Version 9.2.

The analytical process was iterative and commenced during data collection. The detailed field notes, describing the context within which interviews occurred and key themes and concepts, were referred to throughout the analysis. A six-phase process of thematic analysis starting with data familiarisation through reading and re-reading of the interview transcripts and making detailed notes; analysis with initial codes labelled in NVivo; searching for themes by sorting and clustering codes; as analysis continued reviewing themes and relationships between them identified; defining and naming themes before finally producing the written report [39]. Engaging with the data thoughtfully and maintaining a reflexive stance enhanced the quality of the analytic process [40]. Triangulation of research findings within and between interview data, field and conference notes, and with existing research, was used to increase validity of the findings [41]. Interview data from Advocates and KIs was triangulated to gain a broader perspective of the Advocate role and recovery from Obstetric Fistula. The perspectives of the clinical and outreach staff gave additional insights into the opportunities and limitations of the Advocate role.

## 3. Results

Analysis allowed us to generate five themes 1) Fears and Joys of Being an Advocate, 2) Economic Empowerment, 3) Retelling Recovery Stories, 4) The importance of Giving and Receiving Support, and 5) social connections which could be brought together under the central organising concept [42] of Renegotiating Stigma and Social Relationships as an Advocate.

### 3.1 Central organising concept: Renegotiating stigma and social relationships as an advocate

In thematic analyses, themes should coalesce around a central organising concept [42], which for these data was identified as ‘Renegotiating Stigma and Social Relationships as an Advocate’. The influence of both social relationships and the social environment on the recovery process was identified. The importance of an environment facilitating treatment opportunities, which leads to increased social participation and acceptance, was expressed by participants. Increased social integration and power within social interactions due to the advocacy role were described. All Advocates reported social support from; family, husbands or both whilst living with Obstetric Fistula, during treatment and beyond.

#### 3.1.1 Theme 1: Fears and joys of being an Advocate

Themes identified from the findings relating to the person-centered element of recovery; include intrinsic and external factors affecting individual motivation to be an Advocate, challenges encountered by Advocates and the positive effect of social support in sustaining motivation. The potential for financial autonomy from the role, and the positive effect financial incentives had on motivation and/or ability to undertake advocacy activities are described.

All participants described feeling thankful to be well as motivating them to agree to become an Advocate. Appreciation of support and kindness received when they were referred for treatment by HPA, or admitted for surgery at the treatment facility, was articulated as a motivating factor by all Advocates. Empathy for others affected by Obstetric Fistula created a desire in the women to undertake prevention activities and assist others to access treatment.

*“What they did for me made me glad in my heart*. *This made me happy to go and hold those meetings, we are happy to be able to help people get this treatment for this sickness; otherwise, we would have all died*.*” (FA5)*

A concept echoed by a KI, describing the Advocates being motivated by humanitarian feelings. Most Advocates articulated the personal satisfaction they gained from their role.

Not many participants reported an understanding of the training, the advocacy role or what would be expected of them when asked to attend an initial meeting to become an Advocate. Some expressed anxiety at being called for the workshop: including feeling fearful.

*“At first, after the first operation I was there to my people, when they came to come and tell me to go, my papa cried he said sometime they had come to take me and sell me, he told me not to go*. *My auntie told him, ‘leave her, let her go they won’t sell her, they are not those kind of people*.*’ I was also afraid, I cried.” (FA5)*

Despite voicing these initial fears, she later spoke of her enjoyment of the training and advocacy activities and her pride at referring three women with Obstetric Fistula for repair surgery. Motivation to become an Advocate did not appear dependant on a clear understanding or expectation of the role. Most participants described happiness or pride at being asked to participate, despite articulating little knowledge of what to expect.

*“No, I wasn’t worried, because my brother he asked her [Maternal and Child Health Aide], ‘what happened?’ and then she said ‘they picked her to go and train for this problem, to go and advise other people, so this is what made them call her to go and train*. *She is to go to Kamakwie for three days to be trained.’ We were the first people for this work” (FA2)*

#### 3.1.2 Theme 2: Economic empowerment

Only one Advocate reported working while living with Obstetric Fistula. Most described surgical treatment enabling them to return to income-generating activities. Potential economic independence from being an Advocate was described as a motivating factor and perceived as beneficial for recovery. Participants described using the money received from being an Advocate to solve problems, provide for their families and avoid disgrace from unpaid debts, leading to increased self-esteem and independence.

*“Yes, it brought changes, when [field officer] comes, when the month is finished, he brings small money, and I don’t need to borrow again from other people*. *I get happiness, at times I have needed to borrow from people and when he comes, I won’t be disgraced again I can now go and pay” (FA4)*

Most Advocates said they would continue their work in the absence of a monetary incentive. Some had been Advocates from the inception of the project, which did not introduce a financial reimbursement for volunteers until later.

*“At first when we began this work, they didn’t give us anything, but we would walk, we would walk*. *Now later they began to give us something*. *Nothing, but we walked.”*

Despite mainly expressing desires to continue activities in the absence of a monetary reward, in fact a number reported ceasing advocacy activities during the two months prior to the interviews as they had not been receiving a financial incentive or visits from NGO staff (due to project changes).

*“This trip, from the time I came out of the training, I have been doing this work, unless for this past two months*. *This past two months, now I am not able to work because I don’t see anybody [from HPA] now.” (FA3)*

The KI interviews also differed. Some suggested financial motivation was minimal while others, acknowledging that the monetary value of the incentive was small and Advocates desired to help their peers, still considered it a strong motivating factor. This participant voiced concerns that if advocacy activities drew women away from other income-generating activities, this could be detrimental.

*“As most of these people are farmers*. *During the rains, they are all engaged with their farms. If you say they should be going out, they will be using a days or two days job on their farm*. *[The money they receive] It is not enough for the money that they are losing.” (KI2)*

The lack of anticipated financial support to pay for her Caesarean Section (although offered by the treatment facility and not a component of this project) de-motivated one of the advocates so severely she ceased advocacy activities shortly after being trained. She described support from her husband as a factor which had motivated her to continue.

*“All of them, all of them, all of them helped me*. *My man was behind me, even this work, my heart wasn’t in it anymore, but he was always behind me, encouraging me, my man, he talked to me to tell me, encouraging me, let me not be discouraged.” (FA1)*

Most participants described several other difficulties, which at times prevented them from carrying out advocacy activities. Challenges included walking long distances, continuing activities in the rainy season and undertaking advocacy activities whilst pregnant or caring for young children; many spoke of the on-going support from HPA field staff, husbands and family members enabling them to overcome some of the challenges making their role difficult.

#### 3.1.3 Theme 3: Retelling recovery stories

Themes relating to the use of narratives, including Advocates telling their own stories, as a mechanism to define a new social role were identified. Participants also articulated plans for the future representing a crucial element of recovery; moving beyond the trauma to achieve new life goals [27].

The Advocates perceived their role primarily to share information to prevent Obstetric Fistula in their communities, and encourage women already affected to access treatment. The use of storytelling was described as a mechanism for these activities by most participants; apart from one who simply described her role as health work. The concept of storytelling focused on the use of ‘Fatu’s’ story (the picture book received during training) for raising awareness of the prevention, causes, treatment and stigma associated with Obstetric Fistula.

*“This is the same Fatu story no more*. *[.....] When young girls get pregnant when they are not fully grown to give birth, now there Fatu’s problem started; then giving birth again in the house, [......] look Fatu is washing her clothes, they are laughing at her*. *Look her man is saying let Fatu not enter into the room, look at her mate^a^ standing up there. Look where Fatu is lying down it is soaked*. *She says ‘eh, what am I going to do?’ Look they are carrying her [to hospital] Now this is Fatu [with the doctor]” (FA4)*

(In polygamous marriages in Sierra Leone the term ‘mate’ is used to refer to one of the other wives married to a woman’s husband.)

Furthermore, some Advocates and most KIs described the use of personal narratives during advocacy activities.

*“One thing I enjoy about working with them is […] they always set the examples on themselves*. *They say I have been suffering from this! I was ashamed; I was shun out by my family, my mother, my husband*. *Until when I have been repaired now, I can stand proudly and talk to you people, so it is your place to come out rather than making it a secret.” (KI2)*

Incorporating personal stories into their advocacy activities was described by participants as a tool to enhance the credibility of their message, make a connection with others affected and confirm the curable nature of Obstetric Fistula. Most Advocates appeared to find the opportunity to create a new narrative by retelling their own story in their role personally rewarding and validating. The experience of sharing their personal story, which reflects the documented story in the ‘Fatu’ story book, provided a mechanism for instilling hope and a motivation among their peers to either change behaviour to prevent Obstetric Fistula or seek treatment.

Most Advocates articulated plans for the future, these centred on financial security and aspirations for a secure home and access to education for their children. Of the participants with living children, most reported educating children as their primary goal.

*“I want to let my children go to school*, *because I didn’t go to school” (FA1)*

A wish to return to education; in the form of skills training rather than formal education was expressed. Most of the advocates voiced a desire to start a business to help them achieve their personal goals. This desire sometimes stemmed from current or anticipated difficulties in undertaking farm work.

*We had cried for those things*. *So, we said we want work to do, to learn work, because like we who have had operations for a long time, to do farm work, we can only try to manage. But they told us in Freetown not to do hard work, but how what can we do, we don’t have other work*. *[....] If I could have a market, this would look better, I would then not need to go and do [farm] work. (FA2)*

#### 3.1.4 Theme 4: The importance of giving and receiving support

Many participants described dependency on others when living with Obstetric Fistula, most relied heavily on family prior to repair surgery. Some received support from their husbands.

*“My mama, she was the one who took care of me*. *When I was there the whole night, changing pieces [of cloth], my mama would go and wash them; my papa would buy soap for me, to wash those pieces.” (FA7)*

The concept of overcoming dependence, and more importantly being able to actively contribute to family or community life, was described as an important part of moving forward and recovering by several the participants.

Participants described receiving successful surgical treatment coupled with interactions created by the Advocate role, enabling them to claim a meaningful social identity as a role model. Themes describing Advocates receiving and providing peer support were identified.

Most described the Advocate role changing community perceptions of Obstetric Fistula, including the Advocates own perceptions. The experience of gaining the correct understanding of the cause of their fistula for the first time during their advocacy training was reported.

*“No! None of them! They didn’t know, they attributed it to something else*. *At the training we told them that the fistula was as a result of that prolonged labour*. *[…] now they know it was as a result of prolonged labour.” (KI1)*

Most participants described the presence of superstitious beliefs in their communities, including associations between Obstetric Fistula and witchcraft, promiscuity, or marital problems. Several participants reported that the information shared by Advocates and their visibility as community role models dispelled myths, reinforced the curable nature of the condition, supported stigma reduction, and changed perceptions of Obstetric Fistula and the women affected.

*“People have the correct understanding now in the communities I have been visiting; they know it is no witchcraft now and it is because of so, so, so reason*. *Delay in labour, not attending ANC during pregnancy time, this and that, so they know now that this is the cause.” (KI2)*

One Advocate, although describing changing other’s perceptions as challenging, perceived her personal story had the power to influence other’s beliefs.

*“I believe say that because people see me now, where they didn’t see me before, some people are happy, they believe that it is something which is curable*.*” (FA3)*

Advocates spoke of providing peer support to others affected by Obstetric Fistula, such as encouraging disclosure, giving advice and support and facilitating access to and uptake of treatment. KIs perceived the Advocates empathy and understanding enabled them to coax other women into disclosing their condition.

*“They have to tell them, ‘I have been a fistula patient, now I am dry, and I am encouraging anyone in this community that is having this problem, not to shy away*. *I want them to come to me directly, I will not tell anyone’*. *At times they build confidence in them.” (KI3)*

Many Advocates reported finding and referring other women with Obstetric Fistula for treatment. To instil confidence in women affected to come forward for treatment, some would share their own stories and experiences, expressing their ability to facilitate treatment for others as giving them great personal satisfaction.

*“The one I helped; they are all well*. *The other one I took they took her to Freetown, I haven’t seen her, but I heard they had the operation […]*. *I feel fine in my heart because they told me this.” (FA5)*

Sadly, an upsetting experience from identifying a woman with Obstetric Fistula, when the woman died shortly after the participant discovered and referred her was reported.

Receiving support from peers, either during admission for treatment or through being an Advocate was identified as beneficial by several participants, who described feeling more hopeful and less socially isolated because of time spent with others recovering from Obstetric Fistula.

*“This is the time that I began to get peace in my heart, when I came to the training and saw other people as when I was here in the village, I thought I was the only person with this problem*.*” (FA5)*

Advocates spoke of support from their peers giving them the confidence to carry out their role. All KIs described witnessing positive and supportive interactions between Advocates and their peers. Despite this, KIs mainly perceived that the benefits of the project were primarily for the community, rather than for the Advocates themselves.

All Advocates reported they would recommend the advocacy role to other women; encouraging them by describing benefits such as the positive environment of the training, knowledge acquired, the support and money received through participation in the project and the sense of purpose and satisfaction derived from preventing Obstetric Fistula and supporting others to receive treatment.

*“I would tell them to go, let them go, let them learn, this is fine work*. *This is work which will make your heart glad*. *I would advise them say, because they gave you a little money, now let you go and teach people about the problem that you got, let other people not get that problem again*.*” (FA3)*

#### 3.1.5 Theme 5: Social connections

Discrimination, traditional beliefs, and consequences of constant wetness and odour, led to feelings of intense shame for many Advocates, preventing them from participating in normal social interactions and daily activities. They vocalised internalised feelings of shame, even in the absence of poor treatment by family or community members.

*“No, nobody drove me away, it was only me*, *I was ashamed to be near people” (FA2)*

*“I was ashamed, because I would sit down and when I get up my lappa would be all soaked, I would shame, the headmen were there*.*” (FA6)*

Some Advocates described feelings of deep despair upon realising that they had Obstetric Fistula.

*“This is a sickness which discourages people, it breaks your heart* *” (FA4)*

Surgical treatment was identified as more important than their advocacy role in overcoming feelings of shame by some.

*“Not the training, I don’t smell anymore*. *[…] Because me, our own party here, who have been mended here*. *I don’t see anyone who is still ashamed, we walk free.” (FA2)*

Most reported extremely minimal social interaction with people outside their immediate family when living with Obstetric Fistula, describing a dramatic change in the quality and quantity of their social relationships following successful repair surgery.

*“The time when I had the sickness, I had a hot temper*. *When I got the medicine now, now I don’t get angry anymore*. *Even if someone brings a fight to me, I’m not going to make a fight because I am happy now*.*” (FA3)*

All Advocates gave accounts of reduced social connectedness resulting from Obstetric Fistula. Some attributed this to their own feelings of shame and others to receiving poor treatment, ridicule, or discrimination from community members. However, none were completely abandoned, and all were supported by family, mainly their mothers, prior to repair surgery. Even women reporting support from husbands reported significant support from family. Most participants described the kindness and compassion received during their surgical treatment as a significant milestone in their recovery journey.

*“The first happiness I got was the place where I went to be mended; there the goodness began, where they treated me*. *There, they took care of me well, we ate well, they gave me clothes, they gave me transport [to return home].” (FA5)*

All Advocates were married at the time of the study. Some reported their husband left them once they developed Obstetric Fistula, all had subsequently remarried. Most of the Advocates that remained married specifically referred to support from their husband whilst living with Obstetric Fistula. The KIs, acknowledging some men would not leave their wives, described abandonment of women with Obstetric Fistula as the norm. The experience of remarrying while living with Obstetric Fistula, his love for her and her commitment to him was described by this Advocate.

*“No, I didn’t know him before*. *He was with me throughout; he loves me a lot*. *He takes care*. *That makes anything I get; I come with it and give him.” (FA6)*

Some Advocates articulated that the greater financial independence and visible relationships with NGO staff from their role translated into greater power and autonomy within their marriages. A concept echoed by all the KIs.

*“It helped me as I don’t need to borrow again*. *[…] The other time me and my man want to make a fight*. *He wanted to hold me, it wasn’t long after I had the operation and I said I will call [HPA field officer] and then he left me.” (FA4)*

Improvements in the quality of social interactions because of new communication skills developed during their training or advocacy work were mentioned.

*“I was short tempered when I was sick but when I was there doing the work, I got peace*. *I had a way to talk to other people, to let them be well too, now my heart changed” (FA7)*

A difficult and uncomfortable encounter with a group of people who were disbelieving of the information the Advocate shared was also discussed.

Most participants described an exclusion from, or lack of participation in, community life prior to becoming an Advocate and spoke of significant changes since undertaking the advocacy role. Advocates articulated experiencing greater self-confidence; one spoke of taking a leadership role in recent community projects and another reported participation in advocacy activities at national level.

*“When I had the fistula, I didn’t have the mind to go there*. *I didn’t go to school; I didn’t understand anything they didn’t call me. Anything they do now, they call me. Just now they called me, there is work they are doing*. *I have a team of my own I am heading*. *[…] Where they called me to the workshop, now in the town they call me to anything.”*

The KIs also gave accounts of observing Advocates increased self-confidence and improved relationships with community members. However, KIs did not describe the role as a reintegration strategy. Perceiving reintegration strategies meant economic empowerment and the volunteer Advocate role had limited capacity to effectively empower women financially.

## 4. Discussion

The findings presented here provide insight and understanding into the experiences of women affected by Obstetric Fistula whom, after receiving surgical treatment, become Fistula Advocates. The accounts of the KIs provided additional insights into the opportunities and limitations of the Advocate role to those of the Advocates. The four elements of recovery identified in Onken’s 2007 framework: *Person-Centered Elements*, *Re-authoring*, *Exchange-Centered Elements and Community-Centered Elements* [27]. were used to further understand analysis. Onken’s framework [27], enables exploration of the complex relationships illustrated in the interviews between individual recovery from Obstetric Fistula and the social environment the women function within. The Advocate role, situated within the context of a project providing training, ongoing supportive supervision, and financial support, appears to promote individual and environmental change supportive of recovery from Obstetric Fistula; within the limitations created by the voluntary nature of the role.

The findings indicate motivation to be an Advocate is multifaceted; incorporating empathy for others experiencing Obstetric Fistula; psychosocial benefits derived from respect and support from HPA staff, peers, family, and community members; coupled with the satisfaction and sense of purpose gained from undertaking prevention activities or supporting women affected to access treatment. Other studies have reported high levels of mental distress among living with fistula because of long-term social isolation, it therefore understandable they would have high level of empathy for others experiencing similar distress [25]. The findings are comparable to benefits for peer providers in the context of recovery from mental illness identified in existing literature [43–45]. Motivation to carry out the role appeared high, often maintained even under challenging circumstances described by some participants. The main factor negatively impacting motivation appeared to be withdrawal of actual or anticipated financial support. The payment of a monetary incentive (even though small) appears to positively influence participants’ motivation or ability to carry out the advocacy role.

Surgical treatment facilitated a return to income-generation activities for most participants. However, some women reported continuing symptoms were a barrier to carrying out agricultural work and reported a lack of alternatives which hindered income-generation in some cases, reflecting previous research into the experience of women treated for obstetric fistula in rural Tanzania [46]. Money received from participation in the project alleviated dependence, providing Advocates with a much needed sense of contribution to life within the family and wider community [47]. However, KIs perceive the volunteer role has limited capacity to economically empower women. This paper suggests that programmes require careful design to avoid financial dependence on husbands or family being translated into financial dependence on projects with a finite lifespan. Equipping women with funds, training, and support to do business is one economic empowerment strategy responding to needs expressed by women in this and other studies [48]. In mental health services, formally employing service users as peer providers is a mechanism supporting improved earning capacity and employment stability [43].

Reflecting on the findings of this paper, the power of NGO workers is evident. The need for appropriate training, in skills such as psycho-social support, to ensure high quality and sensitive programme delivery is clear. The need for providing psycho-social support is highlighted by a qualitative study looking at women’s experiences of living with OF in the central region of Malawi found women’s lives and social connections were impacted by fear of embarrassment and stigma [49]. The FPP intervention may have benefitted from a mental health specialist; supporting the women to process and recover from trauma. This is also an important ethical consideration for future research in this area. Support for NGO workers working with women who have experienced trauma is also needed.

This study reflects findings from existing research identifying the importance of surgical treatment for physical and mental health recovery and social reintegration [46, 50]. Studies which looked at the experiences of women living with Obstetric Fistula in Uganda and Malawi identify a need for health programmes to focus not only on repair surgery but also on addressing the stigma and social isolation women with Obstetric Fistula experience to improve their quality of life [49, 51]. The analysis of these findings, whilst supporting the importance of repair surgery, suggest being an Advocate enhances the mental health recovery initiated by relief of physical symptoms. Enabling women to gain credibility and new identities as role models by providing community education, defining and sharing their stories [43, 45] which have the potential to reduce stigma and isolation. The interconnectedness of the suggested benefits of the Advocate role and the supportive environment created by the project are difficult to disentangle and require further exploration.

A study by Turan et al. [52] suggests women affected by Obstetric Fistula may be willing community educators but social and economic empowerment is crucial to build the self-confidence required for this role to be effective. Onken [27] identifies two concepts of recovery; overcoming symptoms of the illness itself and the resulting social exclusion. Recovery from Obstetric Fistula incorporates both elements [15, 23]. The importance of addressing the underlying socio-cultural determinants of Obstetric Fistula in addition to providing physical treatment is crucial [23]. Improving the status of women is fundamental to reduce the incidence of Obstetric Fistula and create an environment which offers the social and economic opportunities to support recovery [52, 53].

The aspirations of the participants in this study are comparable to other evidence, describing women focusing on providing schooling for children and planning to do business [46]. The limited range of personal aspirations described may reflect the narrow range of education and employment opportunities accessible to women living in rural areas of Sierra Leone [34]. Striving to create a better life than before the trauma occurred is an element of the recovery process [27]. To gain independence women must have control over decisions about their future [54]. Improving information sharing when inviting participants to volunteer and engaging them as active partners in project planning [6], (a concept demonstrated earlier in the project, when women affected by Obstetric Fistula participated in development of the picture book currently used by Advocates) would mitigate fears voiced by participants and enhance their ability to determine how to invest their time [27].

A significant proportion of the women in this study remained married and received support from family members. These findings support other studies [46, 55, 56] which differ from the body of literature which describes most women being abandoned by husbands and ostracised by communities. A qualitative study of Ugandan men’s experiences of living with a wife suffering from obstetric fistula found they described a conflict when trying to balance the expectations of the hegemonic masculinity present in Uganda and the challenges of living with a wife with Obstetric Fistula [57]. Participants gave accounts of supportive relationships with HPA staff coupled with greater economic independence leading to improved relationships with husbands. The potential impact of the Advocate role on existing relationships is of note as one described a situation where her relationship with NGO staff was protective against domestic violence. Yeakey et al. 2011, [23] describe the positive impact of surgical treatment on, not only women affected, but also their husbands and a subsequent improvement in interpersonal relationships. The importance of portraying an accurate representation of women with Obstetric Fistula, avoiding perpetuating unnecessary stereotypes and stigma has been highlighted [8, 55, 58]. The findings of this study provide further evidence of a spectrum of experiences following Obstetric Fistula.

Participants indicated new or enhanced social interactions, facilitated by peer support and education elements of the advocacy role, supported the reduction of stigma and promoted reintegration [6, 45]. Being with other women affected by Obstetric Fistula during treatment and advocacy training was valued highly by participants, whom described their encounters with others as fostering hope and reducing social isolation [45]. Furthermore, participants identified providing peer support and education led to psychological benefits. They gained satisfaction and a sense of purpose from taking positive action to prevent others from developing Obstetric Fistula, and supporting those affected to access treatment [44]. The literature available on the experiences of peer service providers, suggests benefits for those delivering [45, 59], and receiving peer support [43]. The ability of women affected to support their peers to access treatment is highlighted in existing literature [2, 60]. The importance of providing appropriate training and support to mitigate negative consequences or emotional stress is essential [59].

The accounts of the participants in this study describe Advocates identifying others affected by Obstetric Fistula and facilitating treatment through referral, allaying fears, and encouraging uptake of treatment. The ability of peer providers to reach those needing services but are unaware of, or unable to access them is described by other studies [43, 61]. Although the data for this study was collected in 2012, more recent research looking at maternal health, unmet needs, and barriers to healthcare in rural Sierra Leone following the 2014–1016 Ebola outbreak, indicates that pregnant women continue to experience barriers to care leading to preventable mortality and morbidity including Obstetric Fistula [62]. The study recommends developing links between communities and health facilities to build trust, facilitating peer-driven quality improvement and actively searching for women with Obstetric fistula during community engagement and outreach work so that they can be referred for corrective surgery [62]. The participation of women affected to develop services for Obstetric Fistula prevention, treatment and recovery has been shown to be beneficial, resulting in services which are more sensitive and culturally acceptable [6].

### 4.1 Strengths, limitations, and future directions

This paper adds to the limited qualitative data on Advocates perception of both their role and its influence on their recovery and reintegration following Obstetric Fistula. A strength of this paper is that interview topic guides were informed by a literature review, the researcher’s knowledge and experience of the country context, and discussions with relevant experts including programme managers, field staff and a Sierra Leone Ministry of Health and Sanitation researcher. A longer timeframe and a more diverse sample would potentially generate a deeper understanding of the advocacy role in recovery of women affected by Obstetric Fistula; however, the sample size was appropriate for this study design, reflecting the exploratory nature of the study, other studies on the experiences of women living with Obstetric Fistula [36], and the resource and time constraints of the project [63]. Furthermore, although some time has elapsed since these data were collected and initially analysed, key qualitative methods scholars have previously commented on the benefit of time in qualitative analyses [64]. Women of a variety of ages, who had lived with Obstetric Fistula for different durations, with and without living children were included in the study. However, there was homogeneity in the sample population as all Advocates had previously received surgical treatment, reported being married as young girls, becoming pregnant soon after; and reported no education. To facilitate a high-quality dialogue individual interviews were used, these enabled women speaking three different local languages, to participate without the challenges of interpretation into multiple languages a group setting would have presented. The researcher’s familiarity with the topic, local culture and Krio language facilitated development of rapport during interviews which also supported a high-quality dialogue.

Onken’s ecological framework [27] was used to understand interview data; as it enables exploration of key themes identified in existing literature relating to recovery from Obstetric Fistula, specifically the complex relationship between individual recovery and the social environment.

Formally employing Advocates has the potential to provide meaningful employment and income generation opportunities; whilst accelerating Obstetric Fistula prevention and treatment. Undertaking further qualitative research focusing on the experiences of women affected by Obstetric Fistula providing and receiving peer delivered services and quantitative research to measure the impact on prevention, treatment and recovery is warranted. Further research exploring the impact of providing or receiving peer support on the recovery of women unable to be successfully treated would be useful to inform strategies for this group at particular risk of social isolation.

## 5. Conclusions

The findings, within the confines of the study limitations described, identify the value women affected by Obstetric Fistula place on receiving treatment, gaining financial independence, and playing an active and meaningful role in social life. The importance of a supportive environment to facilitate these achievements is crucial. Overall Advocates perceived their role positively, reporting psychological, social, and economic benefits from their participation in the project. The Advocates described being equipped with funds, training, and support as empowering, which enhanced reintegration to their communities, and enabled them to pass on information to educate other women with Obstetric Fistula to seek surgical treatment. The complexities of recovery from Obstetric Fistula were highlighted and connections drawn between the treatment of physical symptoms, the socio-cultural context and mental health recovery. The role was described to positively influence existing relationships and initiate supportive, empowering social interactions both between women affected by Obstetric Fistula and with NGO staff and community members. The study offers insights into the potential for community-based approaches to facilitate access to treatment for sensitive and stigmatising health problems and support recovery.
